# The Discipline of Suffering

**Published:** 1886-10-30

**Authors:** 


### Words of Consolation.

[Communications for this column should be addressed, "Chaplain," care of the Editor of The Hospital.]

### V.—The Discipline of Suffering.

There are few so perfect in this world as not to need the discipline which only suffering can give to character. Sickness meets most people, either as sinners needing repentance, or as penitents needing discipline. But there are some, whom we call, in our ignorance, poor sufferers, infinitely rich in their privilege, because they suffer as if selected to bear witness for Christ. While we try to sympathise, we wonder why such an unequal share of pain has been allotted to them. We know them to be good Christians. Nay, more: have we not observed how the discipline of sickness has told for good upon them, how meek and resigned they have become, how lovely and refined their characters have grown through much tribulation? Why should God persist in afflicting them? we are inclined to ask, seeing that they need so little discipline compared with most Christians. Ah! we have forgotten, that there is a privilege reserved for some of God's children, in suffering. We have forgotten that He has many secret martyrs in the noble army, besides those who suffer persecution or violent deaths. The end of all long-suffering endurance is not merely that the individual character may be disciplined and perfected, but that the Christian may, through the fiery trial, win victories over Satan for God. Satan is the author of sin, and through sin he has produced sickness and death. But God has mercifully allowed these very swords of Satan, in the hands of the Saints, to be turned against him. The enemy is permitted to afflict faithful souls, only to find them waxing bolder through tribulation, growing stronger in the Spirit, being crucified with Christ. These are already more than conquerors with Him. We may count them happy indeed who endure in such a cause. They are nothing to the outside world but poor sufferers; much to be pitied, perhaps; not by any means to be envied; the last of all to be considered as privileged. How different do they appear to the eyes of the spiritual world? To myriads of excited beholders in the heavenly kingdom, they are conspicuous actors in the terrible drama, which is being rapidly played out, between the kingdoms of light and darkness. Such a calling, because it is so marked with the Cross, is probably the very highest which is possible on this side of the grave.

On the other side of the grave we are assured that there will be more than a corresponding weight of glory. Possibly this may consist, not only in the satisfaction of having triumphed over sin, but in having suffered so much as Christ did, so meekly, so patiently, in filling up the measure of His affliction. "If so be that we suffer with Him, that we may be also glorified together" (Rom. viii, 17). "If we suffer we shall also reign with Him" (2 Tim. ii, 12).

(*To be continued.*)

The Proper Weight of Man.—Professor Huxley asserts that the proper weight of man is 154 pounds, made up as follows:—Muscles and their appurtenances, 68 pounds; skeleton, 24; skin, 10½; fat, 28; hair, 3; thoracic vicera, 3½; abdominal vicera, 11; blood which would drain from the body, 7. The heart of such a man should beat 75 times a minute, and he should breathe 15 times a minute. In 24 hours he should vitiate 1,750 cubic feet of pure air to the extent of one per cent. A man, therefore, of the weight mentioned, should have 800 cubic feet of well-ventilated space. He should throw off by the skin 18 ounces of water, 300 grains of solid matter, and 400 grains of carbonic acid, every 24 hours; and his total loss during that period would be 6 pounds of water, and a little more than 2 pounds of other matter.

